# The Impact of Artificial Intelligence Usage on Affective Work Well-Being: A Self-Determination Theory Perspective

**DOI:** 10.3390/bs16050670

**Published:** 2026-04-28

**Authors:** Chi Zhang, Shuping Chen, Dianru Zhang, Qichao Zhang

**Affiliations:** 1 Mental Health Education Center, Beijing Jiaotong University, Beijing 100044, China; chizhang@bjtu.edu.cn; 2 School of Marxism, Beijing Jiaotong University, Beijing 100044, China; 3 School of Economics and Management, Beijing Jiaotong University, Beijing 100044, China; 4 HSBC Business School, Peking University, Beijing 100871, China; drzhang@stu.pku.edu.cn; 5 Business School, Beijing Information Science and Technology University, Beijing 102206, China; zhangqichao@bistu.edu.cn

**Keywords:** AI usage, affective work well-being, work meaningfulness, work engagement, human–AI collaboration quality, self-determination theory

## Abstract

Despite the growing integration of artificial intelligence (AI) into workplace operations, how AI usage influences employees’ affective work well-being remains underexplored. To address this gap, this study draws on self-determination theory to propose a moderated mediation model. We conducted a three-wave time-lagged survey of 360 employees and our results indicate that AI usage positively predicts affective work well-being, and this relationship is fully mediated through two parallel paths—work meaningfulness and work engagement. Moreover, human–AI collaboration quality positively moderates these indirect effects. Collectively, these findings extend self-determination theory to AI-augmented work contexts, clarify the psychological mechanisms linking AI usage to employee affective well-being, and provide actionable insights for human-centered AI implementation.

## 1. Introduction

The rapid adoption of artificial intelligence (AI) technologies has fundamentally transformed the nature of work across industries. Recent estimates suggest that AI will affect nearly 40% of all jobs worldwide (International Monetary Fund, 2024). While AI’s potential to enhance productivity and streamline workplace processes is increasingly recognized (Lane et al., 2023), its impact on employee psychological well-being remains complex and underexplored. Empirical evidence reveals a mixed picture: some studies demonstrate that thoughtfully integrated AI adoption benefits employee well-being by optimizing work tasks and reducing physical job demands (Valtonen et al., 2025), with measurable improvements in workers’ physical health through reduced job intensity (Giuntella et al., 2025). Conversely, other research highlights concerns about technostress—the strain experienced from increased technology use—job insecurity, and intensified workplace demands (Sharif et al., 2025; Chuang et al., 2025; Jin et al., 2024).

To provide a clear conceptual foundation, we draw on Fisher’s (2014) framework of workplace well-being, which identifies three major components: subjective well-being (comprising job satisfaction, positive attitudes, and affective states), eudaimonic well-being (encompassing engagement, meaning, growth, and intrinsic motivation), and social well-being (involving quality connections and relational satisfaction). Building on this framework, the present study focuses on affective work well-being—the emotional dimension of subjective well-being—as the focal outcome variable. Work meaningfulness and work engagement are conceptualized as eudaimonic mechanisms that explain how AI usage influences employees’ emotional experiences at work. This distinction allows us to conceptually distinguish between employees’ emotional states and their psychological functioning, thereby avoiding conceptual overlap between predictors and outcomes. Drawing on the affective well-being tradition in occupational psychology, we define work-related affective well-being as employees’ subjective emotional experiences and affective states elicited by the psychosocial work environment, which is conceptualized along the dual dimensions of pleasure and arousal, and specifically manifested as four interrelated affective constructs at work: anxiety, comfort, depression, and enthusiasm (Mäkikangas et al., 2007).

Furthermore, although social well-being constitutes an important dimension within Fisher’s framework, the present study centers on individual-level psychological processes triggered by AI usage. As such, relational dynamics are not explicitly modeled, which allows for a more focused examination of the internal motivational mechanisms linking AI usage to affective outcomes.

Despite growing scholarly interest in AI’s workplace implications, three critical gaps remain in the literature. First, existing studies exhibit a strong instrumental bias, prioritizing AI’s efficiency and performance effects while overlooking its profound influence on employees’ emotional and psychological well-being (García-Madurga et al., 2024). This neglect fails to capture the full spectrum of AI’s impact on employees, as psychological well-being is not only an end in itself but also a precursor to sustained performance. Second, the psychological mechanisms linking AI usage to employees’ affective outcomes remain theoretically underdeveloped. While associations between AI implementation and employee outcomes have been documented, few studies have investigated the intermediate psychological processes that explain these effects. Although recent evidence suggests that work meaningfulness and work engagement serve as critical yet understudied mediating pathways (X. Liu et al., 2025; Gemmano et al., 2026; Landells & Albrecht, 2019), no study has systematically examined their combined parallel mediating roles in the AI–well-being relationship. Third, research has paid insufficient attention to human–AI collaboration quality as a boundary condition. The manner in which humans and AI systems collaborate fundamentally shapes employee experiences and outcomes. AI systems enhance employee well-being when they preserve meaningful decision-making opportunities and support psychological needs for autonomy and competence (Passalacqua et al., 2025), whereas systems that eliminate human agency erode motivation and satisfaction (Haefner et al., 2023). Yet the moderating role of human–AI collaboration quality on these well-being outcomes remains largely unexamined empirically.

To address these gaps, this study draws on self-determination theory (SDT; Ryan & Deci, 2017, 2022) to examine how AI usage influences employee affective work well-being. SDT offers a robust framework for understanding how workplace technologies affect well-being by explaining the role of three fundamental psychological needs—autonomy, competence, and relatedness—in shaping motivation and psychological functioning (Deci & Ryan, 2000). The theory posits that social contexts supporting these needs foster intrinsic motivation and well-being, while contexts frustrating these needs undermine both motivation and well-being. As AI fundamentally reconfigures work contexts, SDT provides a theoretically grounded lens for understanding its psychological implications. We develop and test an integrated moderated mediation model to address three core questions. First, does AI usage predict employee affective work well-being? Second, do work meaningfulness and work engagement mediate this relationship, serving as the eudaimonic psychological pathways through which AI affects hedonic well-being? Third, does human–AI collaboration quality moderate these indirect effects, such that high-quality collaboration strengthens AI’s positive influence on both mediators?

The study contributes theoretically by extending SDT to AI-enabled work environments and empirically validating the psychological mechanisms—work meaningfulness and engagement as eudaimonic states—that link technology use to affective well-being outcomes. By positioning collaboration quality as a boundary condition, we move beyond treating AI as monolithic toward understanding how implementation characteristics shape outcomes. Practically, our findings inform organizational AI deployment strategies by identifying design features that preserve employee psychological needs while leveraging technological capabilities.

## 2. Theory and Hypotheses

### 2.1. Theoretical Background

Self-determination theory (SDT) is one of the most empirically validated frameworks for understanding human motivation and well-being (Ryan & Deci, 2017). At its core, SDT posits that humans have three innate psychological needs: autonomy (experiencing volition in one’s actions), competence (feeling effective in one’s endeavors), and relatedness (experiencing connection with others). When work environments support these basic psychological needs, employees experience autonomous motivation—engaging in activities because they are inherently interesting or aligned with personal values. Conversely, when needs are frustrated, employees experience controlled motivation, characterized by external pressures or internal compulsions, which leads to diminished well-being and performance (Gagné & Deci, 2005).

SDT’s distinction between autonomous and controlled motivation proves particularly powerful in organizational contexts. Van den Broeck et al. (2021) conducted a comprehensive meta-analysis revealing that intrinsic motivation most strongly predicts employee well-being, engagement, and reduced counterproductive behavior, while identified regulation (a form of autonomous extrinsic motivation) most powerfully predicts performance and organizational citizenship behavior. Critically, the meta-analysis demonstrated that autonomous motivation consistently outperforms controlled motivation across work-related outcomes, reinforcing SDT’s core proposition that the quality of motivation matters more than its quantity.

This theoretical distinction becomes especially relevant when examining AI’s workplace impact. Technology can either enhance basic psychological needs by eliminating tedious tasks and enabling creative work, or undermine them by constraining choice and imposing rigid procedures. Recent applications of SDT to digital technologies have consistently shown that their effects on well-being depend critically on whether they support or frustrate employees’ fundamental needs for autonomy, competence, and relatedness. Specifically, technology that enhances autonomy through increased discretion promotes positive outcomes, whereas technology that reduces autonomy through excessive monitoring or threatening competence through opacity generates stress and disengagement.

### 2.2. Artificial Intelligence Usage and Affective Work Well-Being

AI usage refers to the extent to which employees integrate AI technologies into task execution within work environments (X. Liu et al., 2025; Wu et al., 2025), encompassing diverse applications including intelligent automation of routine tasks, AI-powered decision support systems, generative tools for content creation, and predictive analytics for workflow optimization. As AI adoption accelerates across all organizational contexts, understanding its psychological consequences for employees’ affective well-being becomes increasingly critical for both theoretical development and practical application.

Consistent with our conceptual framework, affective work well-being, in this study, refers to employees’ subjective emotional experiences and affective states elicited by the psychosocial work environment. Affective work well-being, as conceptualized within Fisher’s (2014) broader framework of subjective well-being, is further specified in this study, drawing on Warr’s model of affective experience (Warr, 1990; Mäkikangas et al., 2007). Specifically, affective well-being is conceptualized along the dual dimensions of pleasure and arousal, manifested as four interrelated affective constructs: enthusiasm, comfort, anxiety, and depression. This conceptualization aligns with the hedonic perspective of well-being, which focuses on pleasure, satisfaction, and positive emotional experiences, distinguishing it from eudaimonic aspects such as meaningfulness and engagement that represent psychological functioning and personal growth (Ryan & Deci, 2000).

Research reveals AI’s complex and often contradictory effects on employee well-being. On one hand, AI can enhance work experiences by automating repetitive tasks, providing intelligent assistance, and enabling employees to focus on more meaningful work. Zhou et al. (2024) demonstrated that while AI generates stress stemming from technological complexity and uncertainty, it simultaneously facilitates employee learning and skill development through human–AI interaction, with learning serving as a critical mechanism linking AI exposure to reduced stress. Likewise, Xu et al. (2025) further documented that AI usage enhances work engagement by increasing psychological availability (the sense of having cognitive and emotional resources to invest in work) and suppressing work alienation. However, AI may also generate adverse consequences. Sharif et al. (2025) found that AI use increases job insecurity and technostress, which in turn impair employee well-being. Moreover, when AI extensively substitutes employees’ core task characteristics—such as taking over central decision-making or creative elements of work—it paradoxically increases alienation and diminishes engagement (Xu et al., 2025). This indicates that not all forms of AI usage produce equivalent outcomes; the degree to which AI replaces versus augments human capabilities proves decisive.

Synthesizing these findings through SDT reveals that AI usage enhances affective work well-being by satisfying employees’ basic psychological needs. When AI expands employees’ discretion and control over work processes, it supports autonomy by freeing workers from rigid routines to engage in more meaningful tasks. When AI provides skill development opportunities and transparent performance feedback, it bolsters competence by enabling employees to master new capabilities and make better-informed decisions. When AI incorporates collaborative features that facilitate human interaction, it fosters relatedness by enhancing rather than replacing social connections in the workplace. Importantly, contemporary organizational AI implementations are typically designed with these need-supportive features in mind, as enterprises recognize that employee acceptance and effectiveness depend on technologies that augment rather than constrain human capabilities. Thus, despite potential downsides, we argue that in contexts where AI serves as an augmentation tool, it satisfies fundamental psychological needs, and it should promote affective work well-being. Based on this reasoning, we propose the following hypothesis:

**H1.**  *AI usage positively influences affective work well-being.*

### 2.3. The Mediating Role of Work Meaningfulness

Work meaningfulness represents the degree to which employees perceive their work as significant, purposeful, and contributing to something beyond themselves. Within the SDT framework, meaningfulness emerges when work activities align with one’s authentic interests and values while simultaneously satisfying needs for competence and autonomy. Empirical evidence demonstrates strong positive relationships between work meaningfulness and job satisfaction, reduced turnover intentions, and enhanced psychological well-being, with meta-analytic findings across studies confirming these associations (Allan et al., 2019). The mediating role of work meaningfulness has been supported in broader organizational contexts (Landells & Albrecht, 2019) and, more recently, in AI-specific settings. X. Liu et al. (2025) found that employee-AI collaboration enhances work engagement through increased perceptions of meaningful work, while Gemmano et al. (2026) showed that AI implementation practices indirectly foster job satisfaction via work meaningfulness. Y. Liu et al. (2026) further found that the AI approach to job crafting positively predicts career sustainability by enhancing work meaningfulness. Taken together, these studies substantiate work meaningfulness as a key eudaimonic mechanism linking AI usage to employee outcomes.

AI usage may influence work meaningfulness through multiple pathways (Valtonen et al., 2025). First, by automating routine and repetitive tasks, AI redirects employee effort toward more complex, creative, and inherently meaningful activities (Zhang et al., 2025). This autonomy-enhancing division of labor allows employees to engage in work that better aligns with their intrinsic interests and values. Second, AI enhances work meaningfulness by augmenting employee capabilities, enabling them to accomplish tasks and achieve outcomes previously beyond their reach (Fügener et al., 2025; Vaccaro et al., 2024). This competence-boosting effect increases employees’ sense of contributing meaningfully. Third, AI systems provide transparent performance feedback with greater objectivity than traditional methods (Biswas et al., 2024), helping employees understand the significance and impact of their contributions more clearly.

Drawing explicitly on SDT, AI usage that supports autonomy through flexible tool adoption, boosts competence through enhanced effectiveness, and clarifies contribution through transparent feedback should increase perceptions of work meaningfulness by satisfying fundamental psychological needs (García-Madurga et al., 2024; Passalacqua et al., 2025). Given the established strong association between meaningfulness and employee affective well-being, work meaningfulness should serve as a critical eudaimonic mechanism through which AI usage influences affective outcomes (Charles-Leija et al., 2023). Specifically, when AI enhances meaningfulness by enabling employees to engage in more significant and valued work, this increased sense of purpose and psychological functioning should translate into greater affective well-being—manifested as higher job satisfaction, more positive emotions, and reduced distress. Thus, work meaningfulness represents a key explanatory pathway linking AI usage to positive affective outcomes. Based on this reasoning, we present the following hypothesis:

**H2.**  *Work meaningfulness mediates the relationship between AI usage and affective work well-being.*

### 2.4. The Mediating Role of Work Engagement

Work engagement represents a positive, fulfilling, work-related state of mind characterized by vigor, dedication, and absorption (Schaufeli & Bakker, 2004). As a eudaimonic construct, engagement captures the quality of psychological functioning and active involvement rather than merely hedonic pleasure (Fisher, 2014). Meta-analytic evidence demonstrates that job and personal resources positively predict work engagement (Mazzetti et al., 2023), and longitudinal studies confirm that organizational-level resources contribute most strongly to sustained engagement over time (Lesener et al., 2020).

Drawing explicitly on SDT’s need satisfaction principles, AI usage may enhance work engagement through several mechanisms. First, AI optimizes task design by automating monotonous activities, redirecting employee effort toward varied and challenging work that better engages skills and interests, thereby satisfying the need for autonomy (Zhang et al., 2025). Second, AI systems provide immediate, data-driven performance feedback with greater transparency than traditional methods (Biswas et al., 2024), supporting competence needs that drive engagement. Third, well-designed AI tools offer employees greater control over work processes, further supporting autonomy through flexible tool adoption and application choices.

AI usage that satisfies autonomy and competence should foster autonomous motivation, which energizes sustained engagement. When AI enhances engagement by enabling employees to work with greater vigor, dedication, and absorption, this heightened eudaimonic state—characterized by optimal psychological functioning and active involvement—should translate into greater affective work well-being. Specifically, employees who are more engaged experience work as more satisfying and emotionally fulfilling, leading to higher positive affect and overall well-being. Thus, work engagement represents a key eudaimonic mediating mechanism linking AI usage to affective well-being outcomes. Based on this reasoning, we present the following hypothesis:

**H3.**  *Work engagement mediates the relationship between AI usage and affective work well-being.*

### 2.5. The Moderating Role of Human–AI Collaboration

Human–AI collaboration refers to the process whereby humans and AI systems work together synergistically to achieve shared goals, with AI providing recommendations or insights and humans guiding and refining AI-generated outputs (Vössing et al., 2022). The quality of human–AI collaboration reflects how effectively the partnership leverages between-task and within-task complementarity through optimal task allocation (automation, augmentation, or human-only execution), calibrated trust, and transparent communication of AI reasoning and uncertainty (Fügener et al., 2025). High-quality human–AI collaboration is characterized by clear division of labor between human and machine teammates, effective complementarity where each party augments the other’s capabilities, adaptive information exchange, and sustained collaborative engagement that enables genuine partnership rather than merely treating AI as a passive tool (Seeber et al., 2020).

Emerging research suggests that the paradigm of human–AI collaboration fundamentally shapes how AI impacts work meaningfulness. Studies examining different interaction paradigms (Supervisory, Advisory, Interactive) reveal that employees find their work more satisfying and meaningful when they directly interact with AI as a teammate, actively participate in decision-making, and remain accountable for outcomes (Hosseini et al., 2024). From an SDT perspective, high-quality collaboration supports autonomy by positioning AI as an enabling rather than controlling tool, and bolsters competence by helping employees clearly perceive their unique contributions alongside AI’s support. Taken together, these mechanisms suggest that collaboration quality determines whether AI usage successfully translates into enhanced work meaningfulness.

Similarly, research on human–AI collaboration dynamics further indicates that collaboration quality also moderates AI’s influence on work engagement. The transparency and explainability that characterize high-quality collaboration create clearer feedback loops, enabling employees to understand both their own contributions and AI’s role in outcomes (Glikson & Woolley, 2020). This transparency supports competence needs by enabling clearer feedback and more consistent progress monitoring, which in turn helps maintain sustained engagement. Trust-based collaboration, shaped by reliable AI performance and transparent system communication, further strengthens sustained engagement by reducing uncertainty and supporting more effective human–AI coordination (McGrath et al., 2025). Furthermore, poor collaboration quality—marked by reduced coordination, communication breakdowns, and inadequate mutual understanding—impairs teamwork and undermines AI’s potential to enhance engagement (Schmutz et al., 2024).

Collectively, these findings suggest that collaboration quality serves as a critical boundary condition determining whether AI usage successfully enhances employee eudaimonic functioning (meaningfulness and engagement), which in turn affects affective well-being. When human–AI collaboration is well-designed and executed, it amplifies AI’s positive effects on both work meaningfulness and engagement. When collaboration quality is poor, these positive effects may be diminished or even reversed. Based on this reasoning, we present the following hypotheses:

**H4.**  *Human–AI collaboration quality moderates the relationship between AI usage and work meaningfulness, such that the positive relationship is stronger when collaboration quality is high.*

**H5.**  *Human–AI collaboration quality moderates the relationship between AI usage and work engagement, such that the positive relationship is stronger when collaboration quality is high.*

**Figure 1:**
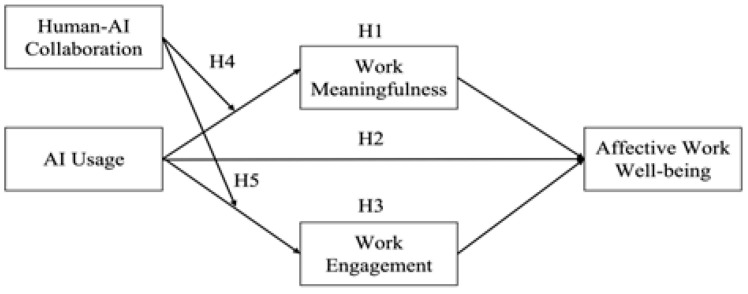
Research Model.

In summary, the proposed research model is depicted in Figure 1.

## 3. Method

### 3.1. Procedure and Sample

Prior to initiating the survey, participants provided informed consent. They were clearly informed of the research purpose (exploring the impact of employees’ AI usage on affective work well-being), the academic nature of the study, the three-wave time-lagged investigation design, the anonymity and confidentiality of all research data, their right to participate voluntarily or withdraw from the survey at any time without any adverse consequences, and the approximate completion time of each survey stage.

This study employed a three-wave time-lagged survey design and collected data via the Credamo online survey platform using a convenience sampling approach. Eligible participants—full-time employees in knowledge-intensive industries who had regular AI interaction at work—were invited to voluntarily complete the questionnaire. The platform supports multi-wave tracking surveys and matched-sample management, making it well suited to the staged matching requirements of this study. A two-week interval between waves was adopted to strike a balance between minimizing common method bias and reducing sample attrition associated with longer tracking periods, while also allowing adequate time for the psychological mechanisms to develop (Dormann & Griffin, 2015; Zhang et al., 2025). At Time 1, the independent variable, the moderator, and the control variables were collected. Two weeks later, at Time 2, the two mediators were measured. After another two-week interval, at Time 3, the outcome variable was collected. This design enhanced temporal separation among the key variables and helped reduce potential interference arising from concentrated same-source measurement. A total of 400 questionnaires were distributed. After excluding responses that failed the attention check and cases that could not be successfully matched across the three waves, 360 valid matched responses were retained, yielding an effective response rate of 90%.

As shown in Table 1, the overall, the sample was demographically well balanced. Respondents were predominantly employees aged 31–35 years, held a bachelor’s degree, and had 6–8 years of work experience. They mainly came from the information technology and finance industries and were primarily engaged in marketing and sales or technical R & D positions, which are characterized by high AI penetration and early adoption, making them suitable contexts for investigating the psychological impact of AI usage. Most respondents reported relatively stable AI usage experience, with daily average AI usage time concentrated between 3 and 5 h. This suggests that the sample as a whole had a relatively strong foundation in AI application, thereby providing appropriate support for research.

### 3.2. Measurement

All key constructs were assessed using measurement scales that have been validated in previous studies. Prior to administering the main questionnaire, a pilot test involving a small sample was carried out to ensure the reliability and suitability of the measurement instruments. With the exception of demographic variables, all items were rated on a five-point Likert scale, and with response anchors adjusted to match the questionnaire’s actual design (frequency anchors for AI usage, agreement anchors for other constructs).

Artificial Intelligence Usage (AI Usage): Measured using the 3-item scale by Tang et al. (2023), assessing the frequency of work-related AI interactions. Sample items include “How often did you proactively initiate work-related interactions with AI?” and “How often did you interact with AI in your work?” Participants rated each item on a 5-point Likert scale ranging from 1 (almost never) to 5 (always). Data collected at T1. Cronbach’s α = 0.857.

Work Meaningfulness: Measured using the 6-item scale from May et al. (2004), evaluating perceived work importance and value. A sample item is “The work I do in this job is very important to me.” Participants rated each statement on a 5-point Likert scale ranging from 1 (strongly disagree) to 5 (strongly agree). Data collected at T2. Cronbach’s α = 0.766.

Work Engagement: We adopted the 9-item UWES-9 scale by Schaufeli et al. (2006), measuring vigor, dedication, and absorption. A sample item is “At my work, I feel bursting with energy.” Participants rated each statement on a 5-point Likert scale ranging from 1 (strongly disagree) to 5 (strongly agree). Data collected at T2. Cronbach’s α = 0.942.

Human–AI collaboration: Measured using the human–machine interaction scale developed by Kong et al. (2023), which assesses employees’ perceptions of the extent to which AI collaborate with them in daily tasks. Sample items include “AI participates in my decision-making process,” “AI participates in my problem-solving process,” and “AI participates in my information identification and evaluation process.” Data were collected at T1. Cronbach’s α = 0.879.

Affective Work Well-being: Assessed with the 6-item scale by Zheng et al. (2015), capturing work satisfaction and meaning. Sample items include “I am basically satisfied with the specific content of my work” and “My work is very interesting.” Participants rated each statement on a 5-point Likert scale ranging from 1 (strongly disagree) to 5 (strongly agree). Data collected at T3. Cronbach’s α = 0.934.

Control Variables: To rule out potential confounding effects, we controlled for gender, age, education, industry, job type, tenure, daily average AI usage time, and AI operation proficiency, as these variables have been shown in prior research to influence employee well-being and work-related behaviors.

### 3.3. Data Analysis

Data analysis was performed using SPSS 31.0 and Mplus 8.3. Descriptive statistics (means and standard deviations) and Pearson correlation coefficients were computed for all study variables using SPSS. Following the guidelines of the American Psychological Association (APA, 7th edition), we report unstandardized regression coefficients (b), standard errors (SE), and *p*-values throughout. A *p*-value less than 0.05 was considered statistically significant.

To assess the discriminant validity of the core constructs, confirmatory factor analysis (CFA) was conducted in Mplus 8.3. The model fit was evaluated using the chi-square statistic, comparative fit index (CFI), Tucker–Lewis index (TLI), standardized root mean square residual (SRMR), and root mean square error of approximation (RMSEA).

Hierarchical regression analysis was then employed to test the direct effect of AI usage on affective work well-being (H1) and the moderating effects of human–AI collaboration quality on the relationships between AI usage and the two mediators (H4 and H5). All regression models included gender, age, education, industry, job type, tenure, daily average AI usage time, and AI operation proficiency as control variables.

To examine the hypothesized mediating effects (H2 and H3) and moderated mediation effects, we applied a nonparametric bootstrap procedure with 5000 resamples. Indirect effects were considered significant if the bias-corrected 95% confidence interval (CI) did not include zero. For moderated mediation, conditional indirect effects were estimated at three levels of human–AI collaboration quality (mean ± one standard deviation).

## 4. Results

### 4.1. Confirmatory Factor Analysis

Given that all data in this study were collected through questionnaires, common method bias could be a potential concern. To mitigate this issue procedurally, we adopted anonymous surveys and collected data in multiple waves. Statistically, we first conducted Harman’s single-factor test, and the results showed that the first unrotated factor accounted for 36.115% of the total variance, which was well below the critical threshold of 40%. However, this test is widely recognized as a rough diagnostic (Podsakoff et al., 2012), so we performed confirmatory factor analysis (CFA) to examine the discriminant validity of the study variables. As shown in the table below, the hypothesized five-factor baseline model demonstrated the best fit to the data, with χ^2^ = 505.104, df = 367, CFI = 0.977, TLI = 0.974, SRMR = 0.040, and RMSEA = 0.032, significantly outperforming all alternative models (Table 2), indicating good discriminant validity among the core variables in this study. To further rule out the influence of common method bias, we applied the unmeasured latent method factor (ULMF) approach. Adding an unmeasured latent method factor to the five-factor model yielded minimal improvement (ΔCFI < 0.02), confirming that common method bias did not seriously threaten the validity of our findings.

### 4.2. Correlation Analysis

Table 3 presents the means, standard deviations, and correlation coefficients for all variables. Overall, AI usage was significantly and positively correlated with work meaningfulness, work engagement, and human–AI collaboration quality. Taken together, the correlation analysis provides preliminary support for the subsequent hypothesis testing.

### 4.3. Hypothesis Testing

Table 4 reports the regression results. AI usage had a significant positive effect on both work meaningfulness and work engagement (Model 1, b = 0.205, *p* < 0.001; Model 3, b = 0.324, *p* < 0.001), supporting the direct paths to the two mediators. Furthermore, the interaction term between AI usage and human–AI collaboration quality had a significant positive effect on both work meaningfulness and work engagement (Model 2 & 4: b = 0.098, *p* < 0.001; b = 0.126, *p* < 0.01), indicating that higher collaboration quality strengthens the positive effects of AI usage on meaningfulness and engagement. Thus, H4 and H5 were supported.

In addition, the results of Model 5, Model 6, and Model 7 show that AI usage had a significant positive effect on affective work well-being, supporting Hypothesis 1. When work meaningfulness was added (Model 6), the direct effect of AI usage became non-significant, while work meaningfulness remained significant (b = 0.347, *p* < 0.001). Similarly, adding work engagement (Model 7) rendered the direct effect of AI usage non-significant, with work engagement being significant (b = 0.156, *p* < 0.01). These patterns are consistent with full mediation via two parallel paths, thereby supporting H2 and H3.

To further examine the mediating effects, this study employed the Bootstrap method, and the results are presented in Table 5. The results show that the indirect effect of AI usage on affective work well-being through work meaningfulness was significant, with an unstandardized coefficient (b) of 0.076 and a 95% confidence interval of [0.036, 0.115], which did not include zero. This indicates that work meaningfulness played a significant mediating role in the relationship between AI usage and affective work well-being. At the same time, after work meaningfulness was included in the model, the direct effect of AI usage on affective work well-being became non-significant, with an unstandardized coefficient (b) of 0.048 and a 95% confidence interval of [−0.058, 0.160], suggesting that work meaningfulness served as a transmission mechanism in this relationship.

In addition, the indirect effect of AI usage on affective work well-being through work engagement was also significant, with an unstandardized coefficient (b) of 0.052 and a 95% confidence interval of [0.014, 0.096], which likewise did not include zero. This indicates that work engagement also played a significant mediating role in the relationship between AI usage and affective work well-being. After work engagement was included in the model, the direct effect of AI usage on affective work well-being became non-significant, with an unstandardized coefficient (b) of 0.072 and a 95% confidence interval of [−0.037, 0.181]. Taken together, these findings indicate that both work meaningfulness and work engagement played significant mediating roles in the relationship between AI usage and affective work well-being. Therefore, Hypotheses 2 and 3 were further supported. The R^2^ values for the regression models in Table 4 ranged from 0.046 to 0.176, indicating small to moderate effect sizes according to Cohen’s (2013) conventions.

To further examine the moderated mediation effect of human–AI collaboration quality, this study employed the Bootstrap method to test the conditional indirect effects at different levels of the moderator. The results are presented in Table 6, showing that when human–AI collaboration quality was at a low level, the indirect effects of AI usage on affective work well-being through work meaningfulness and work engagement were both non-significant, as their 95% confidence intervals included zero. As human–AI collaboration quality increased to the mean level and the high level, both indirect effects became significant, and their effect sizes exhibited an increasing trend. These results indicate that higher human–AI collaboration quality enables AI usage to more effectively enhance employees’ work meaningfulness and work engagement, thereby further improving their affective work well-being. Therefore, the moderated mediation effect of Hypotheses 4 and 5 was supported.

**Figure 2:**
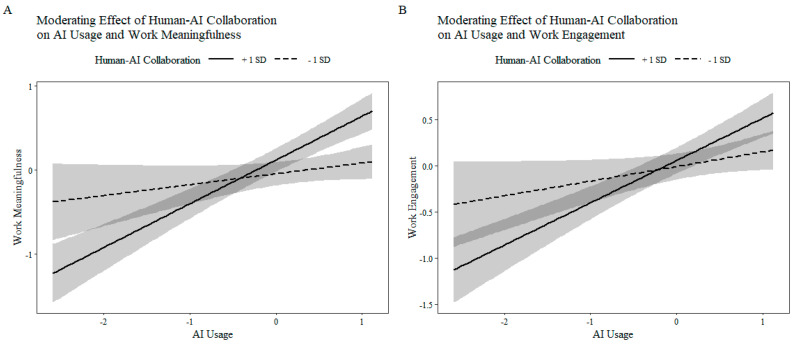
Simple Slope Plots of the Moderating Effects. (**A**) Moderating effect of Human–AI Collaboration on the relationship between AI Usage and Work Meaningfulness; (**B**) Moderating effect of Human–AI Collaboration on the relationship between AI Usage and Work Engagement.

**Figure 3:**
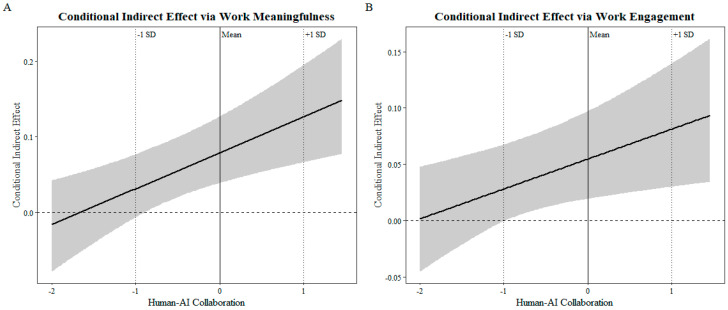
Slope Plots of the Moderated Mediation Effects. (**A**) Conditional indirect effect of AI Usage on Affective Work Well-Being via Work Meaningfulness at different levels of Human–AI Collaboration quality; (**B**) Conditional indirect effect of AI Usage on Affective Work Well-Being via Work Engagement at different levels of Human–AI Collaboration quality.

To provide a more intuitive illustration of the differences in simple slopes for the moderating effect and the changing pattern of the moderated mediation effect across levels of the moderator, this study further plotted interaction effect graphs and conditional indirect effect graphs. Specifically, the simple slope plots were generated based on the estimated regression models and examined how the relationship between AI usage and the mediators changed when the moderator was at low and high levels, thereby visually demonstrating the direction and strength of the moderating effect, as shown in Figure 2. The conditional indirect effect plots were constructed based on the estimated conditional indirect effects at different values of the moderator, with the moderator on the horizontal axis and the conditional indirect effect on the vertical axis, and with confidence intervals included to depict the dynamic trend of the mediation effect across levels of the moderator, see Figure 3 for details The graphical results further indicate that as the level of human–AI collaboration quality increases, the positive effect of AI usage on the mediators becomes progressively stronger. Correspondingly, the conditional indirect effects of AI usage on affective work well-being through work meaningfulness and work engagement also exhibit an increasing trend. When human–AI collaboration quality is low, the indirect effects are weak and non-significant, whereas at the mean and high levels of human–AI collaboration quality, the indirect effects become substantially stronger and reach statistical significance.

## 5. Discussion

Grounded in Self-Determination Theory (SDT; Ryan & Deci, 2017), this study addressed three core questions: (1) Does AI usage predict affective work well-being? (2) Do work meaningfulness and work engagement mediate this relationship? (3) Does human–AI collaboration quality moderate these indirect effects? Using three-wave time-lagged data from 360 employees in knowledge-intensive industries, the results fully supported all proposed hypotheses.

First, our findings demonstrate that AI usage significantly enhances employee affective work well-being, providing empirical support for the “AI empowerment” perspective (Viljakainen et al., 2025; Filippelli et al., 2026). While a considerable body of literature has emphasized the “dark side” of AI—such as technostress and intensified workplace demands (Sharif et al., 2025; Jin et al., 2024)—our results align with a positive counter-narrative: when AI is effectively integrated into workflows, it serves as a supportive resource that streamlines task execution and elevates the overall work experience (Tang et al., 2023). Based on our results, AI usage fosters well-being by automating routine tasks to enhance autonomy, providing data-driven feedback to bolster competence, and preserving essential social interactions to maintain relatedness (Valtonen et al., 2025). This suggests that AI is not inherently detrimental to employee psychology; rather, it functions as a potent resource that cultivates emotional pleasure and arousal in the digital workplace (García-Madurga et al., 2024), depending on how it is designed and implemented. By positioning affective well-being as the primary outcome, this study clarifies the emotional rewards of technological adoption, moving beyond the instrumental performance metrics that have long dominated the field (Lane et al., 2023).

Nevertheless, these positive findings should not be overgeneralized. In contexts where AI extensively substitutes human labor—particularly in low-skill or highly routinized jobs—well-being benefits may diminish or reverse due to job displacement and heightened technostress (Sharif et al., 2025). Our sample was drawn from knowledge-intensive industries, where AI primarily functions as an augmentation tool supporting complex tasks rather than replacing core responsibilities. Under such conditions, the need-satisfying features of AI dominate, leading to positive affective outcomes. Future research should examine contexts where AI’s substitution capacity is higher.

Second, the parallel mediating roles of work meaningfulness and work engagement explicate how AI reconfigures the fundamental relationship between humans and their tasks. Consistent with SDT, AI does not merely change what employees do, but reshapes their perception of role relevance. Specifically, AI usage enhances the perception of work meaningfulness, which in turn fosters affective well-being (Landells & Albrecht, 2019; Gemmano et al., 2026). When AI systems handle routine or low-value drudgery, employees can reallocate their cognitive and emotional resources toward tasks requiring creativity, complex problem-solving, or strategic thinking. This transition enhances the perceived value and significance of their professional contributions (Zhang et al., 2025). This finding aligns with X. Liu et al. (2025), who noted that human–AI collaboration boosts engagement by magnifying the perceived meaningfulness of work. Simultaneously, work engagement serves as a critical conduit; when employees perceive AI as a tool for heightened efficiency, they are more likely to invest energy and passion into their roles. This elevated engagement functions as a psychological catalyst, fostering positive emotional states and long-term flourishing (Mazzetti et al., 2023).

Finally, the moderating role of human–AI collaboration quality reveals that the psychological benefits of technology depend not only on the extent of AI usage but on the effectiveness of the partnership (Haefner et al., 2023). Our analysis suggests that high-quality collaboration functions as both a trust buffer and a competence amplifier. When the collaboration is perceived as reliable, transparent, and supportive, employees are more inclined to trust the technology and integrate it seamlessly into their workflows, thereby maximizing its positive impact on meaningfulness and engagement. Conversely, low-quality collaboration may trigger role ambiguity and threat perception. In such contexts, employees struggle to identify their unique value alongside AI, leading to a diminished sense of purpose and withdrawal from work (Glikson & Woolley, 2020). This underscores that the implementation characteristics of AI systems are the decisive factors in determining whether technology serves as a catalyst for flourishing or a source of workplace strain (Passalacqua et al., 2025).

### 5.1. Theoretical Contributions

First, this study contributes to the growing literature on artificial intelligence in organizations by shifting the research focus from task performance to employees’ psychological experiences and well-being, thereby extending self-determination theory (SDT) to AI-augmented work contexts. Existing studies on AI in the workplace have overwhelmingly concentrated on its impact on productivity, decision quality, and job design, while relatively little attention has been paid to employees’ emotional experiences in AI-enabled work environments. By demonstrating that AI usage can positively influence employees’ affective work well-being, this study expands the understanding of how emerging technologies shape employees’ psychological outcomes. In doing so, this study responds to recent calls for more research examining the human consequences of AI adoption in organizations (Haefner et al., 2023).

Second, by uncovering the psychological mechanisms through which AI usage influences employees’ affective work well-being, this study makes a significant contribution to the field of organizational behavior. Based on Self-Determination Theory, this study identifies work meaningfulness and work engagement as key mediating mechanisms linking AI usage to employees’ emotional experiences. Existing research on AI and well-being either overlooks psychological mechanisms or focuses on a single mediating pathway (e.g., X. Liu et al., 2025, who only focus on work engagement). The results indicate that the value of AI technology lies not only in improving work efficiency but also in its ability to influence employees’ perceptions of work value and meaning, thereby shaping their emotional experiences (Allan et al., 2019; Zhang et al., 2025). Specifically, AI does not affect affective well-being through direct efficiency gains alone, but rather by reshaping employees’ perceptions of work purpose and their active investment in work. By integrating these two pathways, we provide a more comprehensive explanation of the psychological impacts of AI than single-mediator frameworks, filling the research gap in the mechanisms connecting AI to affective outcomes.

Finally, it identifies human–AI collaboration quality as a critical boundary condition, explaining why AI adoption yields heterogeneous psychological outcomes across organizations. Prior research has largely treated AI as an exogenous variable, neglecting how interaction quality shapes employee responses (Haefner et al., 2023). Our findings show that high-quality collaboration amplifies AI’s positive effects on need satisfaction, meaningfulness, and engagement, while low-quality collaboration triggers role ambiguity and threat perceptions (Glikson & Woolley, 2020). This advances the human–AI collaboration literature by positioning interaction quality as a key moderator of psychological pathways, rather than a mere antecedent or outcome. It also addresses the research gap—insufficient attention to boundary conditions of AI’s well-being effects—by demonstrating that AI’s value is context-dependent, not inherent.

### 5.2. Practical Implications

First, organizations should recognize that the adoption of artificial intelligence technologies is not only a technological decision but also a managerial issue that can shape employees’ psychological experiences at work. The findings of this study suggest that AI use can enhance employees’ affective work well-being when it becomes an integral part of employees’ daily work processes. Therefore, managers should move beyond simply introducing AI tools and instead focus on facilitating employees’ effective use of these technologies. For example, organizations can provide training programs that help employees understand how AI systems support their tasks and improve decision-making. By enabling employees to use AI as a supportive work resource rather than perceiving it as a threat or replacement, organizations can better realize the psychological benefits associated with AI-enabled work environments.

Second, in promoting AI adoption, organizations should prioritize employees’ work meaningfulness and work engagement. The results of this study indicate that these two psychological states are important mechanisms linking AI usage to employees’ affective work well-being. Therefore, when designing AI-supported work systems, managers should enable employees to clearly perceive the value and meaning of their work. AI tools should be designed with flexible workflows rather than rigid, one-size-fits-all processes that restrict employees’ autonomous decision-making. At the same time, they should be paired with targeted training programs—focusing not only on technical operations but also on interpreting insights generated by AI to make informed decisions. AI should be configured to automate administrative and transactional tasks rather than social or collaborative activities, freeing up time for face-to-face communication and teamwork. For example, customer service teams can use AI to handle routine inquiries, allowing employees to focus on complex, emotionally intensive interactions that require human empathy.

Third, organizations should prioritize improving human–AI collaboration quality to maximize AI’s psychological benefits, which can be implemented through three key strategies: formally defining the tasks suitable for AI (such as large-scale data analysis and routine forecasting) and those requiring human expertise (such as strategic decision-making and conflict resolution) to clarify human–AI role boundaries and eliminate work ambiguity; clearly explaining AI’s decision-making logic, inherent limitations and potential errors to employees through user-friendly dashboards or special training courses, and building employees’ trust in AI and reducing work uncertainty by enhancing technological transparency; positioning AI as a team partner rather than a work replacement through internal communication, training material dissemination and leadership guidance to foster an organizational culture of human–AI collaboration, and establishing regular feedback mechanisms such as anonymous surveys and focus groups to timely identify and address collaboration pain points including unclear task division and opaque AI outputs.

### 5.3. Limitations and Future Research

This study has several limitations. First, the sample was drawn from knowledge-intensive industries in China, which may limit generalizability. Employees in these sectors typically have higher digital literacy and more frequent AI interaction than those in traditional industries (e.g., manufacturing, agriculture). Future research could expand the sample to include diverse industries and cross-cultural contexts to test the robustness of the proposed model. For instance, individualistic cultures may prioritize autonomy more strongly, potentially amplifying the mediating role of work meaningfulness, while collectivistic cultures may emphasize relatedness, altering the moderating effect of collaboration quality.

Second, the measurement of key constructs relies primarily on self-reported data, which may be subject to social desirability bias (e.g., employees overrating work meaningfulness or collaboration quality). Future research could complement self-reports with objective indicators: for example, AI usage frequency and type could be tracked via system logs, collaboration quality could be assessed through observer ratings or task performance data, and affective well-being could be measured using physiological indicators or experience sampling methods.

Third, this study focuses on general AI usage without distinguishing between AI types or functions. Different AI applications (e.g., decision-support AI, creative AI, embodied robots) may have distinct effects on need satisfaction and well-being. For example, creative AI may enhance work meaningfulness by augmenting creativity, while surveillance-oriented AI may undermine autonomy. Accordingly, future research could explore the differential impacts of AI types and functions. Additionally, individual differences (e.g., AI self-efficacy, openness to technology, personality traits) may shape how employees perceive and respond to AI. Incorporating these variables as moderators could provide more tailored insights for AI implementation.

## 6. Conclusions

Against the backdrop of the widespread application of AI in the workplace, this study, grounded in self-determination theory (SDT), empirically explored the influence mechanism and boundary condition of AI usage on employees’ affective work well-being by constructing a moderated mediation model. The results confirmed that AI usage has a significant positive impact on employees’ affective work well-being, with work meaningfulness and work engagement playing parallel mediating roles in this relationship, and human–AI collaboration quality positively moderating the above indirect effects.

Theoretically, this study expands the application of Self-Determination Theory in AI-enabled work contexts and enriches the research on the psychological mechanisms of AI’s impact on employee well-being by clarifying the dual mediating and critical moderating effects. Practically, it provides targeted guidance for enterprises to implement human-centered AI applications, specifically, designing AI systems to support employees’ basic psychological needs, focusing on activating the mediating roles of work meaningfulness and work engagement, and prioritizing the improvement of human–AI collaboration quality to maximize the psychological benefits of AI. These measures can foster a virtuous cycle between human–AI collaboration and employee well-being.

## Figures and Tables

**Table 1:** Descriptive Statistics.

Variable	Category	Frequency	Percentage	Variable	Category	Frequency	Percentage
GI	Female	181	50.30%	JT	Technical R & D	121	33.60%
	Male	179	49.70%		Marketing & Sales	164	45.60%
Age	30 years and below	51	14.20%		Administration & HR	72	20.00%
	31–35 years	269	74.70%		Operations Management	3	0.80%
	36–40 years	39	10.80%	Tenure	2 years and below	19	5.30%
	41 years and above	1	0.30%		3–5 years	75	20.80%
EL	High School and Below	1	0.30%		6–8 years	157	43.60%
	Technical Secondary School	3	0.80%		9–11 years	79	21.90%
	Junior College	27	7.50%		12 years and above	30	8.30%
	Bachelor’s Degree	304	84.40%	Industry	Finance	104	28.90%
	Master’s Degree and Above	25	6.90%		Information Technology	180	50.00%
DUT	Less than 1 h	53	14.70%		Manufacturing	13	3.60%
	1–3 h	72	20.00%		Culture & Education	31	8.60%
	3–5 h	170	47.20%		Public Administration	31	8.60%
	More than 5 h	65	18.10%		Others	1	0.30%

Notes: N = 360; GI = gender identity; EL = educational level; DUT = daily average AI usage time; JT = job type. The following is consistent.

**Table 2:** Confirmatory Factor Analysis Results.

Model	χ^2^	df	CFI	TLI	SRMR	RMSEA
Five-factor model	505.104	367	0.977	0.974	0.040	0.032
Five-factor + method factor	392.392	338	0.980	0.977	0.031	0.021
Four-factor model	945.386	371	0.904	0.895	0.086	0.066
Three-factor model	1791.550	374	0.762	0.742	0.132	0.103
Two-factor model	3425.579	376	0.489	0.448	0.181	0.150
One-factor model	3846.211	377	0.418	0.374	0.186	0.160

Note: χ^2^ = Chi-square; df = degrees of freedom; CFI = Comparative Fit Index; TLI = Tucker-Lewis Index; SRMR = Standardized Root Mean Square Residual; RMSEA = Root Mean Square Error of Approximation.

**Table 3:** Means, standard deviations, and correlations among study variables.

Variable	Mean	SD	1	2	3	4	5	6	7	8	9	10	11
1. GI	1.497	0.501	-										
2. Age	1.972	0.511	−0.120 *	-									
3. EL	3.969	0.45	0.105 *	−0.016	-								
4. Industry	2.189	1.207	0.061	0.009	0.067	-							
5. JT	1.881	0.746	−0.109 *	0.320 **	0.089	0.016	-						
6. Tenure	3.072	0.985	−0.135 *	0.591 **	−0.008	−0.009	0.395 **	-					
7. DUT	2.686	0.935	−0.112 *	0.011	−0.056	0.018	0.018	0.064	-				
8. AU	3.792	1.079	−0.088	0.123 *	0.006	−0.029	0.044	0.079	0.432 **	-			
9. WM	3.993	0.628	−0.000	0.102	0.058	0.023	0.118 *	0.084	0.101	0.336 **	-		
10. WE	3.434	1.044	−0.024	−0.004	0.036	−0.042	−0.069	0.048	0.114 *	0.318 **	0.191 **	-	
11. AWB	3.313	1.159	0.034	0.011	−0.120 *	−0.079	0.030	−0.033	−0.108 *	−0.178 **	0.016	−0.036	-
12. HAC	3.599	0.953	−0.140 **	0.080	−0.030	−0.026	0.070	0.013	0.027	0.137 **	0.260 **	0.190 **	0.158 **

Notes: GI = Gender; EL = Education Level; JT = Job Type; DUT = Daily AI Usage Time; AU = AI usage; WM = work meaningfulness; WE = work engagement; AWB = Affective work well-being; HAC = Human–AI collaboration; The following is consistent. * *p* < 0.05, ** *p* < 0.01. SD = standard deviation.

**Table 4:** Results of Regression Analysis.

Variable	Work Meaningfulness		Work Engagement		Affective Work Well-Being		
	Model 1	Model 2	Model 3	Model 4	Model 5	Model 6	Model 7
(Intercept)	3.993 *** (0.031)	4.014 *** (0.031)	3.434 *** (0.052)	3.462 *** (0.052)	3.599 *** (0.050)	3.599 *** (0.048)	3.599 *** (0.048)
GI	0.043 (0.064)	0.030 (0.063)	−0.014 (0.107)	−0.029 (0.106)	−0.236 * (0.102)	−0.263 ** (0.098)	−0.247 * (0.099)
Age	0.034 (0.077)	0.021 (0.075)	−0.153 (0.129)	−0.166 (0.128)	0.144 (0.123)	0.115 (0.118)	0.149 (0.119)
EL	0.057 (0.070)	0.073 (0.069)	0.103 (0.118)	0.114 (0.118)	−0.051 (0.112)	−0.027 (0.109)	−0.020 (0.110)
Industry	0.014 (0.026)	0.024 (0.026)	−0.027 (0.044)	−0.017 (0.043)	−0.011 (0.041)	−0.006 (0.040)	0.004 (0.040)
JT	0.075 (0.046)	0.082 (0.045)	−0.148 (0.077)	−0.136 (0.077)	0.078 (0.074)	0.038 (0.071)	0.086 (0.072)
Tenure	0.008 (0.041)	0.006 (0.040)	0.115 (0.069)	0.110 (0.068)	−0.080 (0.065)	−0.071 (0.063)	−0.085 (0.064)
DUT	−0.032 (0.037)	−0.028 (0.037)	−0.036 (0.063)	−0.032 (0.062)	−0.046 (0.060)	−0.029 (0.057)	−0.034 (0.058)
AU	0.205 *** (0.032)	0.189 *** (0.033)	0.324 *** (0.054)	0.299 *** (0.055)	0.124 * (0.052)	0.077 (0.053)	0.099 (0.053)
WM						0.347 *** (0.082)	
WE							0.156 ** (0.049)
AWB		0.045 (0.027)		0.030 (0.046)		0.136 ** (0.043)	0.147 *** (0.043)
HAC		0.098 *** (0.024)		0.126 ** (0.041)			
R^2^	0.131	0.176	0.119	0.142	0.046	0.123	0.103
Adj. R^2^	0.111	0.152	0.099	0.118	0.024	0.098	0.078
F Value	6.603 ***	7.435 ***	5.908 ***	5.796 ***	2.111 *	4.882 ***	4.021 ***

Notes: * *p* < 0.05, ** *p* < 0.01, *** *p* < 0.001. Values in parentheses = unstandardized regression coefficient; R^2^ = coefficient of determination; Adj. R^2^ = Adjusted R^2^.

**Table 5:** The mediating roles of work meaningfulness and work engagement.

Parameters	Effect	Estimate	Boot SE	95% CI	
				Boot LLCI	Boot ULCI
AU → WM → AWB	Indirect	0.076 ***	0.020	0.036	0.115
	Direct	0.048	0.056	−0.058	0.160
	Total	0.124 *	0.052	0.024	0.227
AU → WE → AWB	Indirect	0.052 *	0.021	0.014	0.096
	Direct	0.072	0.055	−0.037	0.181
	Total	0.124 *	0.052	0.024	0.227

Notes: Bootstrap resampling was used to test the mediation effects. An effect is considered significant when the 95% confidence interval does not include zero. Indirect refers to the indirect effect, Direct refers to the direct effect, and Total refers to the total effect. LLCI and ULCI denote the lower and upper limits of the 95% confidence interval, respectively. * *p* < 0.05, *** *p* < 0.001.

**Table 6:** Bootstrap Test for Moderated Mediation Effects.

Mediated Path	Moderator Level	Indirect Effect	Boot SE	95% Boot LLCI	95% Boot ULCI
AU → WM → AWB	Mean − SD	0.026	0.017	−0.006	0.060
	Mean	0.066 ***	0.019	0.029	0.104
	Mean + SD	0.105 ***	0.031	0.046	0.166
AU → WE → AWB	Mean − SD	0.024	0.016	−0.003	0.058
	Mean	0.047 *	0.019	0.012	0.086
	Mean + SD	0.069 *	0.028	0.018	0.127

Notes: Moderator levels were set at one standard deviation below the mean, the mean, and one standard deviation above the mean. Boot SE = bootstrap standard error; LLCI = lower limit of confidence interval; ULCI = upper limit of confidence interval. * *p* < 0.05, *** *p* < 0.001.

## Data Availability

The data presented in this study are available upon request from the corresponding author.
